# Resequencing of *Rosa rugosa* accessions revealed the history of population dynamics, breed origin, and domestication pathways

**DOI:** 10.1186/s12870-023-04244-5

**Published:** 2023-05-04

**Authors:** Fengqi Zang, Yan Ma, Qichao Wu, Xiaolong Tu, Xiaoman Xie, Ping Huang, Boqiang Tong, Yongqi Zheng, Dekui Zang

**Affiliations:** 1 State Key Laboratory of Tree Genetics and Breeding, Laboratory of Forest Silviculture and Tree Cultivation, Research Institute of Forestry, Chinese Academy of Forestry, Beijing, 100091 P. R. China; 2 College of Forestry, Key Laboratory of State Forestry Administration for Silviculture of the Lower Yellow River, Shandong Agricultural University, Tai’an, 271018 Shandong P. R. China; 3 State Key Laboratory of Genetic Resources and Evolution, Center for excellence in Animal Evolution and Genetics, Kunming Institute of Zoology, Chinese Academy of Sciences, Kunming, 650223 Yunnan P. R. China; 4 Kunming College of Life Science, University of Chinese Academy of Sciences, Kunming Yunnan, 650204 P. R. China; 5 Shandong Provincial Center of Forest and Grass Germplasm Resources, Jinan, 250102 P. R. China

**Keywords:** *Rosa rugosa*, Genome resequencing, Population structure, Population genetics

## Abstract

**Background:**

*Rosa rugosa* is a shrub that originated in China and has economic and ecological value. However, during the development of *R. rugosa*, the genetic background was chaotic, and the genetic structure among different wild populations was unclear, as well as wild and cultivated accessions. Here, we report whole-genome resequencing of wild and cultivated *R. rugosa* accessions.

**Results:**

A total of 19,041,284 SNPs were identified in 188 *R. rugosa* accessions and 3 *R. chinensis* accessions by resequencing. Population genetic analysis revealed that cultivated and wild groups were separated very early. All *R. rugosa* accessions were divided into 8 categories based on genetic structure: (1) Weihai, Yantai, and Liaoning category, (2) Jilin category, and (3) Hammonasset category (above three are wild); (4) traditional varieties, (5) hybrids between *R. rugosa* and *R. chinensis*, (6) Zizhi Rose, (7) Kushui Rose, (8) hybrids between *R. rugosa* and *R. multiflora*. We found that the heterozygosity and genetic diversity of wild accessions were generally lower than those of cultivated individuals. The genes that were selected during cultivation were identified, and it was found that these genes were mainly related to environmental adaptation and growth.

**Conclusions:**

The Jilin population was the oldest population and later migrated to Liaoning and then migrated to Yantai and Weihai by sea regression in the Bohai Basin. The Hammonasset naturalized population probably originated from the Jilin population and then experienced separate differentiation. The long-term asexual reproduction pattern of *R. rugosa* decreased genetic diversity in the wild population. During *R. rugosa* cultivation, the ancestors of the Jilin population were involved in breeding traditional varieties, after which almost no wild individuals were engaged in breeding. However, in recent decades, cross breeding of *R. rugosa* started the utilization of wild germplasms. In comparison, some other species play important roles in variety formation. Few genes related to economic traits were selected, suggesting no directional domestication in the *R. rugosa* cultivation process.

**Supplementary Information:**

The online version contains supplementary material available at 10.1186/s12870-023-04244-5.

## Background

*Rosa rugosa* is a member of *Rosa* in the Rosaceae family. It has been cultivated for a long time in China for ornamental and edible purposes and has high medicinal value [1–3]. Petals of *R. rugosa* have a unique aroma, from which essential oils can be extracted and widely used in beauty products and perfumes. Moreover, it is highly resistant to drought, barren and cold conditions and has a wide range of adaptability to harsh natural environments [4]. In its native distribution areas, *R. rugosa* plays an indispensable role in stabilizing the ecological environment in coastal areas [5].

In China, the natural distribution area of *R. rugosa* is along the Tumen River of Jilin Province, Zhuanghe of Liaoning Province, and Yantai and Weihai of Shandong Province. It was introduced to Europe in the 18th century as breeding parents and then introduced to America in 1845 [6, 7]. Since then, it has become a naturalized plant in America [8]. However, it is still an endangered wild plant in China. In recent years, the distribution area has been shrinking. It was listed in the Red Book of Chinese Plants in 1992 [9] and listed as a second-class National Key Protected Wild Plant in 2021.

In terms of cultivation and breeding, some traditional *R. rugosa* varieties have been cultivated in China due to their horticultural and medicinal value; the main areas are Pingyin County in Shandong Province, Miaofengshan Mountain in Beijing, and other Central Plains areas. In addition, Kushui Rose, a hybrid type, is also cultivated in Kushui District in Gansu Province [10, 11]. In recent decades, wide new varieties of *R. rugosa* have been developed by crossing with *R. chinensis*, *R. multiflora* and *R. davurica* [12]. The phylogenetic origin of cultivated *R. rugosa* remains to be determined due to its complex background and chaotic history.

Human selection, cross, and geographical isolation have led to the diversity of phenotypes and genotypes. As we know now, domesticated categories vary in flower colours and types. The wild category varied much less than the domesticated category, but there were still some small variations in growth habits, leaves, thorn density, and seed weight [13]. In previous studies, domesticated roses (*Rosa*) underwent frequent hybridization and backcrossing, in which several wild species were involved in early breeding. *R. rugosa* has the advantage of resistance to an adverse environment, which can provide excellent resources for *Rosa* breeding. Germplasms from origin centres are of extraordinary value in exploring and preserving genetic and phenotypic diversity for breeding applications, as they determine the continued ability of plant breeders to develop new high-quality varieties [14]. Therefore, excavating the genome of *R. rugosa* genetic resources is imperative.

In 2021, the genomes of wild *R. rugosa* were sequenced [15, 16]. The final assembled genome sequence size was approximately 407.1 Mb, the contig N50 was 2.85 Mb, and the scaffold N50 was 56.6 Mb. More than 98% of the assembled genome sequences were anchored to seven pseudochromosomes (402.9 Mb). The genome contained 37,512 protein-coding genes, of which 37,016 genes (98.68%) were functionally annotated and 206.67 Mb (50.76%) were repetitive sequences [15]. Previous research set the stage for genetic studies in *R. rugosa*.

The current lack of exploration and utilization of genomic data is a major limiting factor for modern breeding. With the massive increase in throughput of next-generation sequencing technology, an increasing number of plant genomes have completed complete whole-genome assembly, and major crops have successively undergone population resequencing [17–19].

Whole genome sequencing can help quickly detect a large number of polymorphic loci. Population expansion and contraction were studied based on polymorphic loci groups. Then, relations between different groups and gene exchange between wild populations and cultivated varieties were analysed. Researchers can speculate on the origin and propagation path of species and the biological processes related to events that adapt to the environment and then find the key genes involved in domestication. Some resequencing studies of *Arabidopsis thaliana* focused on environmental adaptation and explored biological processes associated with adaptation events [20]. Research on rice has revealed that African rice experienced regional adaptation divergence at these loci [21]. The study of *Pyrus* spp., *Juglans regia*, *Malus domestica*, and other species focused on the patterns of divergence, dispersal, and independent domestication, revealed the formation history of different groups of species, explored the spread from the origin to the suitable habitat, and explored the genes related to selection and domestication [22–24]. The analysis of genome resequencing and chloroplast sequencing results of *Prunus mume* revealed the transmission routes along the Yangtze River basin system and the Pearl River basin system [25]. Whole-genome resequencing of *Acer yangbiense* germplasm revealed the factors contributing to the current distribution fragmentation and endangered status, which provided basic information and conditions for further conservation [26].

The main objectives of this study were to assess the genetic diversity, population structure, and genetic relationships between wild and cultivated *R. rugosa* populations in different regions of China. Through genetic variation analysis, we explored the origin, migration route, evolutionary relationship, domestication mechanism, and selected genes of *R. rugosa*.

## Results

### Genome variations in *R. rugosa*

A collection of 188 *R. rugosa* and 3 *R. chinensis* accessions were sequenced (Additional file 1: Table S1.). After filtering out low-quality reads, 839.24 Gb of clean data was generated, with an average of 4.39 Gb per sample; the average effective depth for our dataset was 8.31-fold, with an average mapping rate of 92.37% coverage of the *R. rugosa* reference genome (Additional files: Table S2, Table S3).

The quality of sequencing data was high (Q30 > 92.62%), and the average mapping read ratio was 97.89%. A total of 19,041,284 SNPs were detected from 188 samples, with conversion (Ts) \/conversion (Tv) ratios between 1.785 and 2.205.

**Fig. 1:**
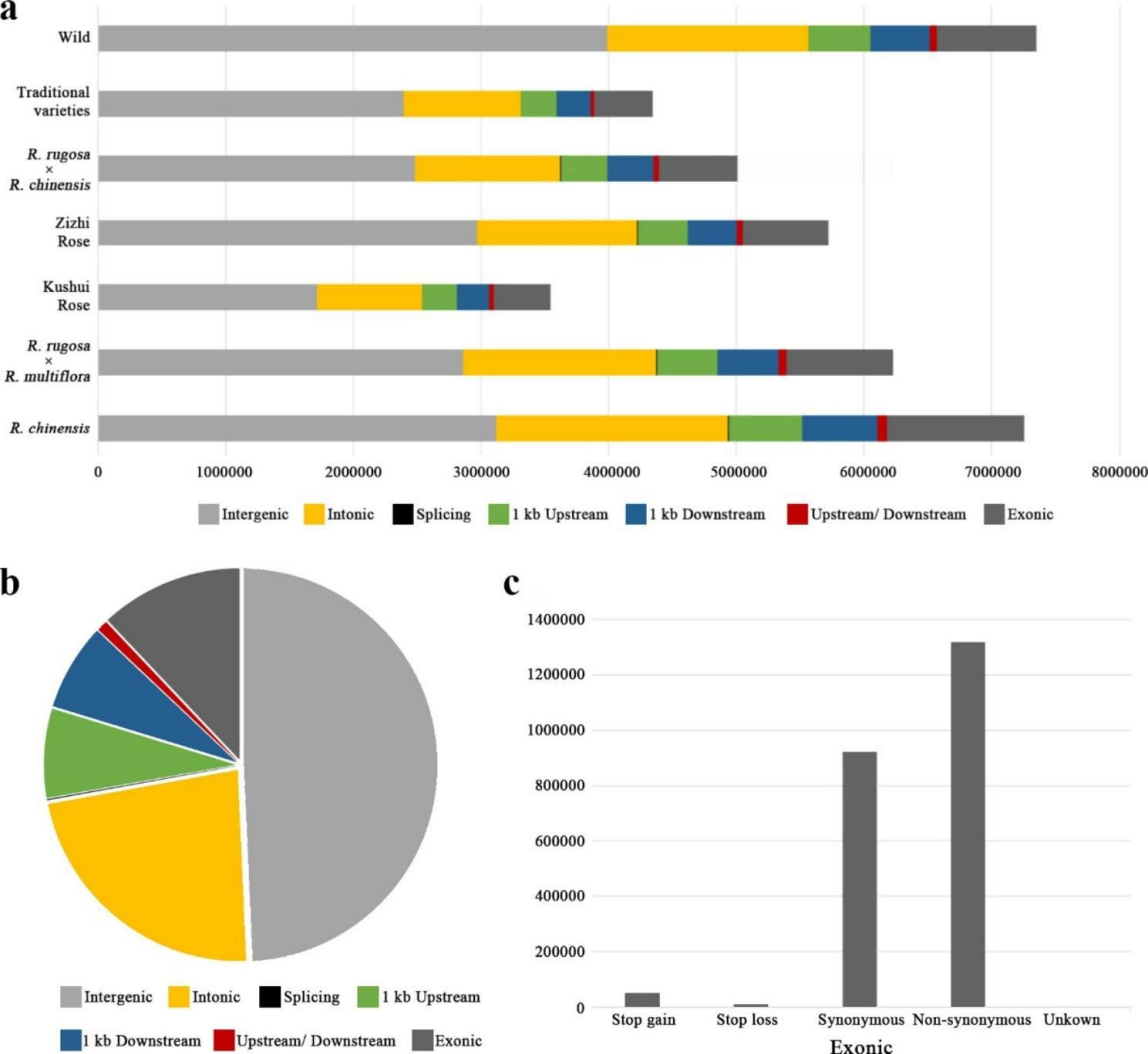
Distribution and categories of SNPs in the genomes of 188 *R. rugosa* and 3 *R. chinensis* accessions. (**a**) Distribution of SNPs of different groups. (**b**) Distribution of all SNPs. (**c**) Distribution of exonic SNPs of all accessions

There were 7,348,719 SNPs in wild *R. rugosa*, accounting for 38.8%. The highest SNPs in cultivated *R. rugosa* were that in hybrids between *R. rugosa* and *R. multiflora* (6,229,599, 32.7%), followed by hybrids between *R. rugosa* and *R. chinensis* varieties (5,00,6429, 26.3%) (Fig. 1a). About 49.28% of the SNPs occurred in intergenic regions, and 22.87% occurred in intronic regions (Fig. 1b). There were 919,094 synonymous coding mutations and 1,317,005 nonsynonymous coding mutations in SNPs of the coding regions (Fig. 1c, Additional files: Table S4, Table S5). Wild germplasm has a highly diverse gene pool, which contains valuable genetic resources for improving *Rosa* cultivation.

### The relationships between wild and cultivated populations based on phylogenetic analysis

**Fig. 2:**
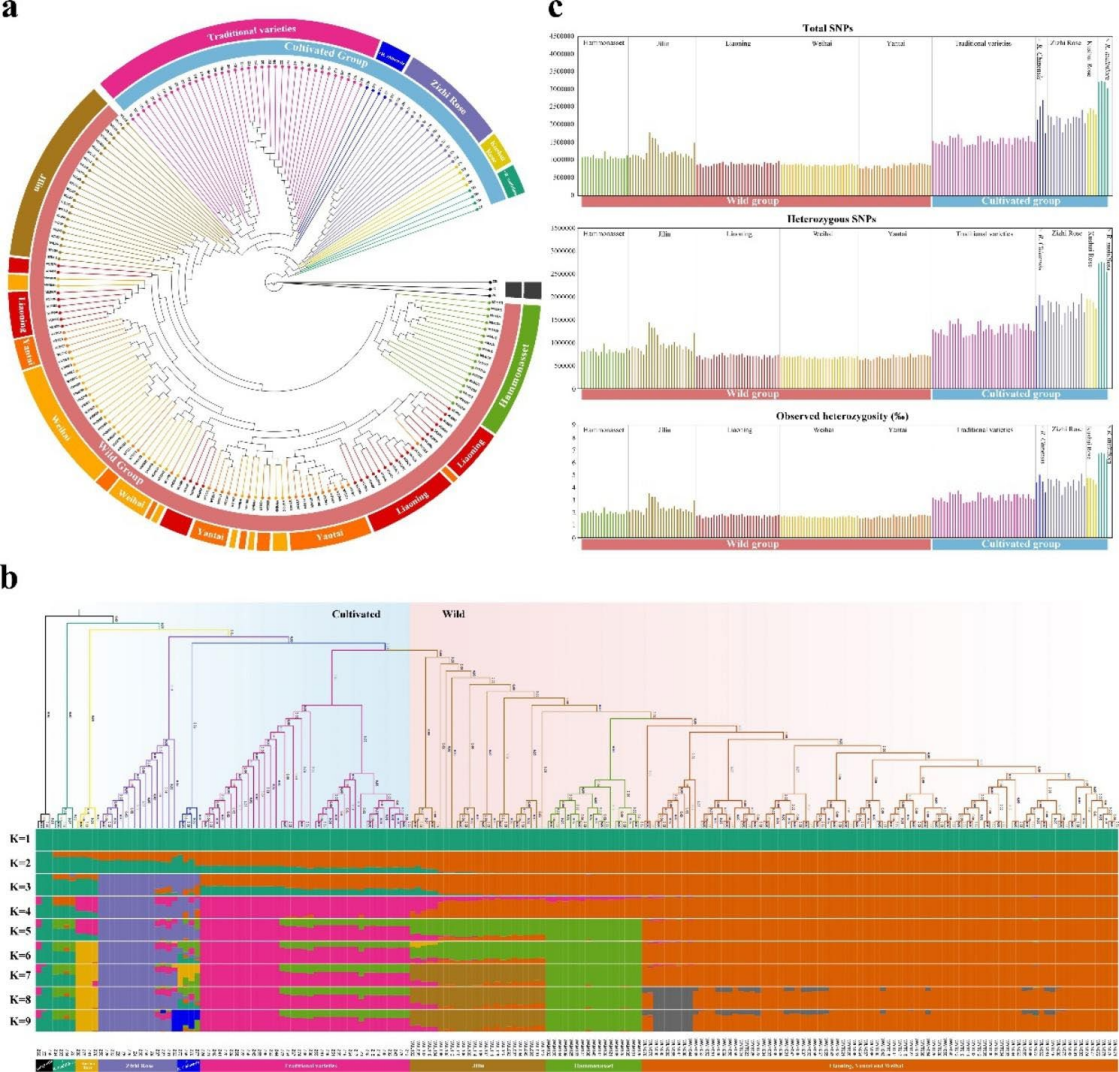
Population structure and phylogeny of *R. rugosa*. (**a**) Phylogenetic tree of 188 wild and cultivated *R. rugosa* and 3 *R. chinensis*. (**b**) Genetic structure. (**c**) Comparison of genetic diversity among different populations of *R. rugosa*

We constructed neighbour-joining trees (NJ tree) using two methods, 56,2663 SNPs at 4-fold degeneracy loci SNPs and 19,041,284 genome-wide SNPs, to study the phylogeny of wild *R. rugosa* and cultivated *R. rugosa*. The taxonomic groups are basically the same in both cases (Fig. 2a, Additional file 14: Fig. S1). According to the phylogenetic tree analysis, three varieties of *R. chinensis*, Z55, Z2, and Z60, were the first to be separated as an outgroup. It was then clear that among *R. rugosa* accessions, the cultivated populations were distinguished from the wild populations at an early stage. They were classified as the wild group and cultivated group, respectively.

Among the wild group, the Jilin population was the first to divide and could not cluster into one clade, but many single clades appeared. The distinguished clade was the Hammonasset population (naturalized in Hammonasset Beach, Connecticut, USA), which suggested that this population may have originated from Jilin. The last part is the Liaoning and Shandong populations (Yantai, Weihai), most of which are mixed. However, some accessions in the Liaoning population form a clade of their own, slightly different from other accessions. Shandong and Liaoning populations had a mixed genetic relationship with common ancestors, which may be related to the transgression, regression, and geological changes of Shandong Peninsula and Liaodong Peninsula.

Among the 63 cultivated accessions, five clades can be clearly seen. The closest clade related to the wild population was traditional varieties, mainly cultivated in Shandong and Beijing. The relationships suggested that traditional varieties shared a recent common ancestor with the Jilin population. Other varieties derived from artificial crosses with related species could also gather together well according to their parental origins. The gathered accessions of hybrids between *R. chinensis* and *R. rugosa* from Shandong Province were the closest to the traditional varieties. Then, the gathering clade was the varieties from Pingyin, Shandong Province, which were hybridized between *R. rugosa* and *R. davurica* (commonly known as Zizhi Rose). These varieties are now widely cultivated in Shandong and neighbouring areas. The accessions from Gansu, all clustered into a clade, were believed to have come from a cross between *R. rugosa* and *R. sertata* (commonly known as Kushui Rose). The last part was the *R. rugosa* hybrid crossed with *R. multiflora*. We divided them into five subgroups based on this. Interestingly, individuals in each artificial cross subgroup had some morphological commonalities. Hybrids between *R. chinensis* and *R. rugosa* and Kushui Rose tended to have smooth, less wrinkled, and hairy leaves, while the twigs of Zizhi Rose were purplish red.

We performed STRUCTURE analysis to assess the proportion of ancestral germplasm in each accession to clarify the genetic history and structure (Fig. 2b). When K = 2, *R. chinensis* (outgroup) showed a completely different structure from the other groups. When K = 3, the genetic structure of traditional varieties was significantly different from that of other cultivated groups but more similar to that of wild *R. rugosa*. According to the minimum coefficient of variation (CV) error value (Additional file 15: Fig. S2), K = 8, all samples were divided into 9 categories, representing the best model of all samples. When K = 8, it was obvious that the genetic structures of wild *R. rugosa* populations in Shandong and Liaoning were similar and could be classified as one group. However, the Jilin and Hammonasset populations were divided into different categories, and the cultivated groups were the same as the five subgroups we expected.

Previous reports indicated that the ice age sanctuaries of *R. rugosa* were in Yantai and Weihai in Shandong Province, as well as in Jilin Province, suggesting that the wild population in Liaoning may have migrated from Shandong [27]. The new evidence suggested that Liaoning populations originated earlier and then migrated to Shandong, and then there was frequent communication between Shandong and Liaoning. In addition, the Hammonasset naturalized population and traditional varieties are more similar to the wild *R. rugosa* distributed in Jilin; cultivated varieties may not be geographically closely related. After its introduction, *R. rugosa* diverged independently in the Hammonasset of America.

Principal component analysis (PCA) of *R. rugosa* samples showed little significant clustering between cultivated and wild *R. rugosa*, which may be due to the lack of long-term directional selection. Most cultivars were selected by spontaneous mutants (Additional file 16: Fig. S3).

### Genetic diversity and population differentiation analysis based on SNPs

The nucleotide diversity and heterozygosity of wild individuals were lower than those of cultivated individuals.

Using sequencing datasets, we observed the average number of variations and nucleotide diversity of each accession in *R. rugosa* populations. Overall, the heterozygous SNPs and heterozygosity of accessions in the wild population were lower than those in the cultivated population (Fig. 2c, Additional file 4: Table S4). We compared the genetic diversity of naturally distributed wild *R. rugosa* populations. Among the wild populations, the Shandong population (including Yantai and Weihai) accessions’ genetic diversity was the lowest (heterozygous SNPs ranged from 609,154 to 763,572, and observed heterozygosity ranged from 1.496–1.876‰); the Liaoning population was second lowest (heterozygous SNPs ranged between 638,806 − 785,870, and the range of the heterozygosity observed was 1.569–1.930‰); and the highest was the Jilin population (heterozygous SNPs ranged from 753,019 − 1,447,165, and observed heterozygosity ranged from 1.850–3.555‰). Combined with phylogeny, wild *R. rugosa* in Jilin originated the earliest and experienced multiple differentiation in history, so it had more diverse genetic information than other wild populations.

We calculated π values for each kind of *R. rugosa* to measure the degree of nucleic acid diversity within different groups (Additional filen6: Table S6). Hybrids between *R. rugosa* and *R. multiflora* (π = 6.753 × 10^− 3^, θ_W_ = 6.429 × 10^−^3) had the highest genetic diversity. The lowest genetic diversity was observed in the wild group (π = 2.085 × 10^− 3^, θ_W_ = 3.192 × 10^− 3^) and traditional varieties (π = 2.942 × 10^− 3^, θ_W_ = 2.394 × 10^− 3^). According to Tajima-D, we considered that the wild population observed heterozygous loci were less than the expected value, and the frequency of rare alleles increased, indicating that the wild group has undergone directional selection or group expansion. We speculated that wild *R. rugosa* had undergone natural selection throughout its history. In addition, hybrids between *R. rugosa* and *R. chinensis* had similar results to the wild population.

Generally, cultivated populations should have lower genetic diversity than wild populations due to artificial selection. However, unlike other plants, the genetic diversity of *R. rugosa* cultivated varieties was higher than that of wild populations. We considered that this phenomenon might be due to its asexual reproduction mode in the wild, while interspecific hybridization often occurs in cultivation.

Genetic differentiation among populations.

*F*_*ST*_ (F-statistics) is a indicator based on whether the actual frequency of genotypes in a population deviated from the genetic balance (Hardy-Weinberg equilibrium) for measuring the degree of population differentiation. According to Wright’s definition, the *F*_*ST*_ value more than 0.25 indicates a high level of genetic differentiation [28]. To study the degree of genetic differentiation among different groups, the *F*_*ST*_ between each group was calculated for evaluation (Additional file 7: Table S7).

The *F*_*ST*_ value between traditional varieties and the wild group (*F*_*ST*_=0.166) was the smallest in all cultivated groups. The values of hybrids between *R. rugosa* and *R. chinensis* varieties (*F*_*ST*_= 0.311), Zizhi Rose (*F*_*ST*_= 0.296), Kushui Rose (*F*_*ST*_= 0.412), hybrids between *R. rugosa* and *R. multiflora* (*F*_*ST*_= 0.409), *R. chinensis* (*F*_*ST*_=0.571) were significantly higher than that of traditional varieties. The genetic relationship between traditional varieties and the wild group was the closest.

The *F*_*ST*_ value between traditional varieties and the wild group was less than 0.25, indicating that them were not completely divided; while the *F*_*ST*_ values between other cultivated groups and wild group were more than 0.25, suggesting a high degree of genetic differentiation.

Since all the cultivated groups except traditional varieties were interspecific crosses between *R. rugosa* and other plants of *the Rosa* genus, this result was consistent with our expectations. The results were also consistent with the previous phylogenetic tree and population structure analysis.

### Population dynamics of *R. rugosa*

**Fig. 3:**
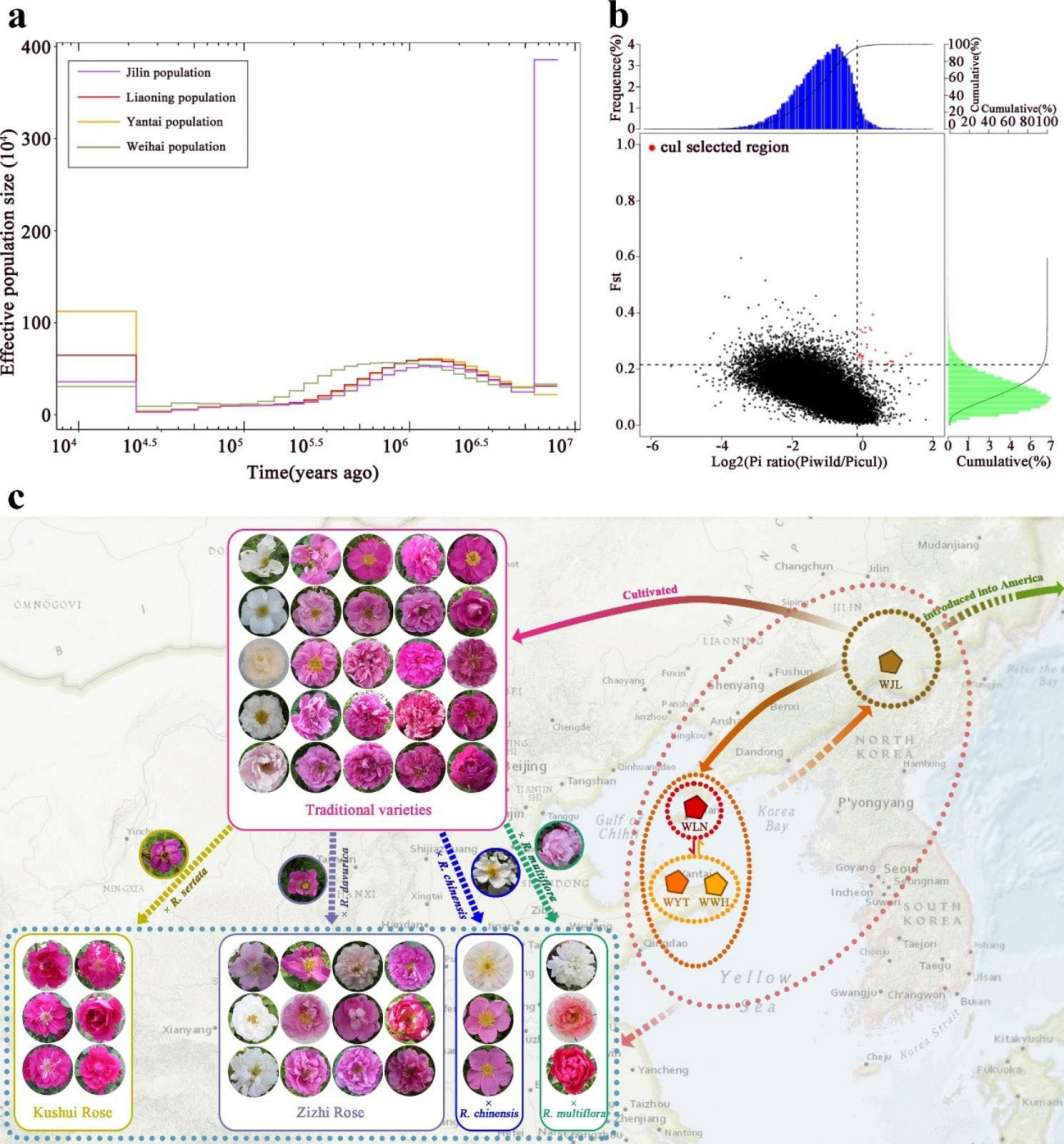
Historical evolution and cultivar formation of *R. rugosa*. (**a**) Historical population size dynamics of *R. rugosa*. (**b**) Selected genes in *R. rugosa*. (**c**) Development routes of *R. rugosa* wild populations and formations of cultivated varieties

To explore the genetic history of *R. rugosa* populations, we estimated the effective population sizes at different periods in history using MSMC software (Fig. 3a, Additional files: Fig. S4, Fig. S5). The results showed that all wild populations had similar historical dynamics, experienced population expansion from species differentiation until approximately 3 million years ago, began to shrink until approximately 1 million years ago, and then began to expand slowly until approximately 30,000 years ago. This roughly matches the geological history of the ice age [29]. It expanded during the interglacial period, contracted during the glacial period, and then expanded after the glacial period. The Jilin population was slightly different from that of other populations, having an extremely large effective population size until approximately 6 million years ago, and then the population size shrank sharply. According to previous studies, species of *Rosa* diverged approximately 6 million years ago, and *R. rugosa* and *R. chinensis* diverged between approximately 5.3 and 6.6 million years ago [15, 16]. This sharp population contraction may have been the result of *R. rugosa* speciation. From this, we can also infer that the Jilin population is the oldest of the existing wild *R. rugosa* populations.

We detected the gene exchanges using the value of BABA-ABBA to test gene flows between different populations (Additional file 8: Table S8). There was significant gene flow between the traditional varieties and other cultivated populations. In contrast, the genetic contribution of wild populations to Kushui Rose and hybrids between R. rugosa and R. multiflora, but less contributed to the traditional varieties. The results of the BABA-ABBA model mainly agreed with previous analyses of genetic structure.

No run of homozygosity (ROH) (> 1 M) was detected in accessions of all wild populations, indicating that few inbreeding and bottleneck effects were observed (Additional file 9: Table S9). Populations that have experienced bottlenecks will have a shorter time to trace their ancestors, and it is easier to find common ancestors. Our phylogenetic analysis showed that there was also no recent common ancestor. Therefore, it was also confirmed that wild populations did not experience bottleneck events in their history.

### Domestication analysis and selected genes

We scanned the regions of the genome with the greatest variation in allele frequency *F*_*ST*_ and genetic diversity (π-log ratio) to detect selected regions. Owing to the complex and partly unresolved demographic history of these populations, it is difficult to define a strict threshold that distinguishes true sweeps from regions of homozygosity caused by drift. Therefore, we used two strategies to detect selected regions.

First, the top 5% highest log2 (θπ·wild/θπ·Cultivated) and top 5% highest *F*_*ST*_ were used to find 31 regions related to selection domestication (Fig. 3b, Additional file 10: Table S10). We examined the candidate genes related to acclimation on the chromosome in the region with the greatest variation in the *R. rugosa* genome and annotated these genes with GO function. With 2 hard-to-annotate genes manually curated and removed, 39 candidate genes were detected, most of which were related to environmental adaptability, including genes in the pathways of “heat acclimation”, “metal ion binding”, “cell wall Organization”, related to biological stress “defence response to bacterium, incompatible interaction”, and genes regulating growth and development (Additional file 11: Table S11). Among them, *FORGETTER 1* is a heat-adaptation-related gene that underwent positive selection during domestication [30].

In addition, 39 regions were identified by the top 0.1% highest *F*_*ST*_ (Additional file 12: Table S12). Afterwards, a total of 52 genes were detected and annotated (Additional file 13: Table S13). GO annotation and classification of these selected genes showed that many of them were related to stress response. For example, “defence response to fungus” and “defence response to bacterium” were related to biotic stress; “response to heat” and “response to oxidative stress” were related to abiotic stress; “jasmonic acid mediated signalling pathway” and “auxin-activated signalling pathway” were related to hormone regulatory pathways. In addition, 4 genes related to flora development were identified among the selected genes. One was a *putative transcription factor of the MADS-MIKC family*, one was a *HERK* gene, and two were *CONSTANS-like* genes.

## Discussion

To elucidate the genetic route of *R. rugosa*, we resequenced the genomes of 125 wild accessions and 63 cultivated accessions. This is the first study to examine the genetic structure, phylogenetic relationships, and domestication history of wild versus cultivated *R. rugosa*.

A previous chloroplast sequencing study elucidated that the genetic diversity of wild *R. rugosa* was low and that it may have multiple glacial refugia, such as Yantai and Weihai in Shandong and Hunchun in Jilin [27]. In the study of edible rose (*R. rugosa* and other species), the origin of modern cultivated edible rose varieties was complex, and there may be several wild species, including *R. rugosa*, involved in the formation of edible rose germplasm in China [31]. On the basis of previous studies, we tried to explore the historical rules of migration and distribution of wild *R. rugosa* and the relationship between wild populations and current dominant varieties.

Analysis of population structure showed that wild *R. rugosa* is mainly divided into three categories: Weihai, Yantai, and Liaoning category, Jilin category, and Hammonasset category. From the population structure and phylogenetic tree, it can be inferred that the wild *R. rugosa* in Hammonasset was from Jilin, and genes of other species were introduced in the differentiation process. On the other hand, the wild *R. rugosa* populations in Jilin and Hammonasset had the closest relationship with cultivated groups, suggesting that the Jilin population may have been more involved in the cultivation and domestication history of *R. rugosa*. Meanwhile, the genetic structures of *R. rugosa* from Weihai, Yantai, and Liaoning were similar. Since the late Pliocene, the continuous subsidence of the basin and the uplift of the surrounding mountains have isolated the Bohai Basin and exposed the land. According to previous research, there was a large regression event in the Bohai Sea approximately 37,000 years ago, when the sea level dropped, the land was exposed, and the Shandong Peninsula and Liaodong Peninsula became geographically linked [32]. These may lead to frequent gene exchange among the wild *R. rugosa* populations in these regions during the regressions of the sea. After the regression event, the sea level rose again, and the two peninsulas were separated again. The populations of Liaoning, Yantai, and Weihai separated again and began to develop. This is similar to the results of previous studies on the *R. rugosa* chloroplast haplotype.

In cultivated accessions, hybrids between *R. rugosa* and *R. multiflora* were first separated, followed by Kushui Rose and Zizhi Rose, which is related to how close their parents are to *R. rugosa*. *R. multiflora* belongs to Sect. *Rosa* in the classification. *R. sertata* and *R. davurica*, both belonging to Sect. *Cinnamomeae* were more closely related to *R. rugosa* (Sect. *Cinnamomeae*). In our study, the genetic structures of Zizhi Rose and Kushui Rose were relatively simple and stable compared to those of other cultivars. We thought this was due to the two subgroups’ parental origins being quite single. Although hybrids between *R. rugosa* and *R. chinensis* showed a later divergence from *R. rugosa* traditional varieties on the evolutionary tree, their genetic background was more complex. We speculated that the genetic background of modern rose was relatively complex, with hybridization and backcrossing between different species.

It is also worth noting that, unlike other species, the wild and cultivated populations of *R. rugosa* diverged very early. For example, in the cultivation study of Chinese apricot, it could be clearly seen that the cultivated varieties came from wild populations in different distribution areas. The results were the same for peaches and walnuts [23, 33]. Combined with the results of gene flow analysis, we speculated that the traditional cultivated *R. rugosa* varieties were domesticated from the wild population in Jilin. Since then, there has been little involvement of wild germplasms in breeding traditional varieties. However, wild germplasms were involved in the breeding of hybrid cultivars, especially Kushui Rose and hybrids between *R. rugosa* and *R. multiflora*.

According to the above discussion, we proposed the conjecture of the genetic introduction route of *R. rugosa* (Fig. 3c). Wild *R. rugosa* probably originated in Jilin and then spread to Liaoning. The Liaoning population spread to the Shandong Peninsula through the marine regression event in the Bohai Basin, forming the Yantai and Weihai populations. We speculated that the origin of the traditional varieties was also ancient individual cultivation in Jilin Province. In the history of later breeding traditional varieties, there were long periods when wild germplasms were not involved. However, many hybrid varieties bred in recent years had genetic contributions to wild germplasm, indicating that the utilization of wild germplasm resources has been strengthened in recent years. However, the genetic contribution of wild germplasms to many hybrid varieties bred in recent years indicated that the utilization of wild germplasm resources has been strengthened in recent years. In addition, the record of Kushui Rose may be a natural hybrid of *R. rugosa* and *R. sertata*, which was supported by our results [10].

In our study, we found that the SNP diversity and heterozygosity of wild *R. rugosa* were lower than those of cultivated varieties, which was very rare in previous studies. Positive selection and population bottlenecks occurred during the domestication of peach, which resulted in a decrease in genetic diversity [33]. Wild soybean has a higher level of genetic diversity than cultivated soybean [34]. Resequencing studies of watermelons have shown that the nucleotide diversity of *Citrullus colocynthis* and *C. amarus*, which are biased towards the wild form, is much greater than that of *C. mucosospermus*, *C. lanatus*, and landraces [35]. The same result was found in *Prunus persica* and *Ziziphus jujuba* [36, 37]. In addition, in the study of apricots cultivated in China and those cultivated in the West, scholars believed that the higher genetic diversity of Chinese apricots was due to frequent gene exchange [38]. In general, traditional cultivars were domesticated from wild types. In this process, cultivars experience a certain degree of bottleneck events, and some genes are selected, resulting in low heterozygosity and genetic diversity. However, our results were not consistent with this, and we speculated for two reasons. When sampling wild *R. rugosa*, we found that wild individuals often reproduce asexually in the form of tillering (Additional file 19: Fig. S6). This has been confirmed in previous studies that wild populations of *R. rugosa* in Europe and coastal areas of China are mostly asexual [39]. Moreover, we found that the distribution of wild *R. rugosa* was fragmented, and habitat fragmentation and discontinuity led to the loss of genetic variation in small populations, resulting in a reduction in population genetic diversity. The second was that cultivated populations had introgression of other related *Rosa* species, and it was known that *R. davurica, R. sertata, R. multiflora*, and *R. chinensis* were involved in the formation of cultivated varieties. In addition, *R. rugosa* is a self-incompatible plant [40]. In other words, in the breeding process of cultivated *R. rugosa*, in addition to the selection of specific plants such as bud mutation, *R. rugosa* must be crossbred for seed reproduction. Therefore, we speculate that the heterozygosity of wild *R. rugosa* is lower than that of cultivated *R. rugosa*, which may be because wild *R. rugosa* is mostly expanded by asexuality, resulting in lower genetic diversity, while cultivated *R. rugosa* is mostly crossbred or even interspecific crossbred, resulting in higher genetic diversity.

In the estimation of effective population size, we found that there was little sharp contraction of *R. rugosa* population size recently, and no large-scale inbreeding or bottleneck events were detected. This was similar to the recent expansion of wild *R. rugosa* populations in studies of chloroplasts. Furthermore, *R. rugosa* in Europe, America and other areas are considered to be invasive plants. Therefore, we speculate the main reason of that *R. rugosa* endangered in China was that the habitat was destroyed by human activities, which led to a lack of population continuity. Based on previous records of wild *R. rugosa* populations and on-the-spot investigations, we found that many of the original recorded wild populations have now disappeared. With the development of the coastal economy, the coastal habitat has been destroyed, resulting in the sudden disappearance of many small populations [41]. In addition, we found that the genetic structure of *R. rugosa* was relatively simple and had low nucleotide diversity. In the wild, sexual reproduction is mainly carried out by insect pollination, and the efficiency is low.

In previous studies, we usually found selections of genes related to the economic shapes of the plant in cultivated populations. Resequencing of soybeans revealed some traits selected for domestication and modification, such as *LPD1*, a gene associated with oil content [34]. A genome-wide association study of tartary buckwheat identified several candidate genes for important agricultural traits, *FtUFGT3* and *FtAP2YT1*, associated with flavonoid accumulation and grain weight, respectively [42]. In the breeding process of *Gossypium barbadense*, a series of genes affecting the development of the cotton fibre pathway were selected [43]. Compared with other species, there were fewer selected genes in cultivated *R. rugosa*, and their functions were more related to environmental resistance and growth regulation. This was somewhat different from related studies in other species. Many selected genes in cultivated crops and ornamental economic plants are more or less related to phenotypic traits. In the phenotype, although the majority of the double flowers in cultivated *R. rugosa* were rarely reflected in the resequencing analysis of cultivation and breeding. Otherwise, phenotypic differences between wild *R. rugosa* populations were very small. In the study of Chen et al., *R. rugosa* was found to have more genes related to floral development than *R. chinensis*, suggesting that *R. rugosa* retained many more genes for floral-related traits [16]. In our previous studies of *R. rugosa* genome, we also found that many genes of *R. rugosa* were enriched in the metabolic pathways of terpenoids, benzenes and flavonoids, and these genes may be related to the colour and fragrance of flowers [15]. Whereas in our present study only four genes related to floral development were identified. We speculated that the reason may be related to its simple breeding process. *MADS-box* family genes are involved in plant growth and development, flower transformation, floral meristem determination, male/female gametophyte development, fruit development and maturation, and somatic embryogenesis [44–47]. Studies on *MIKC*^*C*^*-type bo*x genes of rose have found that these genes played a potential regulatory role in controlling flowering time and floral organogenesis [48]. *HERK1* may play a role in pollen tube development [49]. *CONSTANS-like* genes may be the key genes for photoperiodic floral formation [50]. These selected genes indicated the beginning of an improvement bottleneck and highlighted important issues in the cultivation process of *R. rugosa*. That is, in the current cultivation process, there was little emphasis on economic traits related to selection, such as flowering and flower colour. This may provide some ideas for the future cultivation of *R. rugosa* varieties.

## Conclusion

In this study, we resequenced 188 samples of *R. rugosa* and 3 *R. chinensis* samples. The historical dispersal routes of *R. rugosa* populations and the relationship between the existing varieties and the wild populations were mapped based on phylogenetic analysis. The expansion and communication of wild populations throughout history were largely influenced by geological changes in the Bohai Basin. After the breeding of the early wild species, there were few wild accessions involved in the cultivation history of *R. rugosa*. We demonstrated that the genetic diversities of *R. rugosa* in the wild were lower than those in the cultivated populations, which was presumed to be due to the *R. rugosa* own breeding system and interspecific hybridization. We analysed the existing problems in *R. rugosa* cultivation and found that the current *R. rugosa* cultivation did not focus on economic traits, which provided a direction for future breeding.

## Materials and methods

### Sampling information and genome resequencing

In this study, 188 samples of *R. rugosa* were collected from the wild population and germplasm collection nursery (Additional file 1: Table S1). We defined 2 groups: Wild group, 105 wild collections from China and 20 from Hammonasset Beach, America; Cultivated group, 63 domesticated collections from main cultivation areas in China. Three *R. chinensis* samples (Additional file 1: Table S1) were selected as outgroups.

We adopted delicate leaves for DNA extraction by the modified CTAB method [51]. The library was prepared using 1 µg gDNA template according to TruSeq DNA Sample Preparation Guide (Illumina, 15,026,486 Rev.C). For each sample, two paired-end libraries (500 bp) were constructed according to the manufacturer’s protocol and sequenced on Illumina novaseq6000 sequencing platform with a PE150 read length.

### Data quality control and reference genome comparison

We conducted several quality control criteria to ensure that the reads were highly reliable. (1) Reads with < = 10% unidentified nucleotides (N); (2) Reads with < = 50% bases having phred quality < 20. (3) Removing reads with > 10 nt aligned to the adapter, allowing ≤ 10% mismatches [52]. Burrows-Wheeler Analyser (v0.7.17) with the parameter ‘mem -t 4 -k 32 -M’ was used for comparison with the reference *R. rugosa* genome (accession number GWHALOL00000000 of CNCB-NGDC Genome Warehouse) [15]. SAMtools (v0.1.19) software was used to convert to BAM format (settings: view -bS -t), and PCR copies were removed using the samtools command “rmdup” [53].

### SNP detection and annotation

The haplotype caller function of GATK (version 4.1.9.0) was used to obtain mutation sites and screen for low-quality mutations [54]. The criteria used to filter the raw SNPs were “QD < 2.0 || FS > 60.0 || MQ < 40.0 || QUAL < 30 || MQRankSum < -12.5 || ReadPosRankSum < -8.0 || SOR > 3.0”. Polynucleotide polymorphisms were ignored, and loci containing SNP markers had to be present in at least 90% of individuals. SNPs of 5 bp near the gap were filtered out. SNP had a minimum depth of 570 and a maximum depth of 5132. A total of 19,041,284 SNPs were detected from 191 samples (188 samples of *R. rugosa* and 3 samples of *R. chinensis*) for subsequent analysis.

### Population structure analysis

TreeBeST (http://treesoft.sourceforge.net/treebest.shtml) built 100 bootstrap replicates, and an NJ tree was constructed using the p-distance matrix [55]. We performed principal component analysis (PCA) using PLINK (v1.90b6.20). ADMIXTURE (v1.3.0) was used to estimate the genetic ancestry of each sample with the default parameters, specifying 2–9 hypothetical ancestral populations.

### Gene flow and historical population size detection

The maximum likelihood method implemented in TreeMix (v1.13) was used to infer gene flow between different populations [55]. First, we infer the maximum likelihood (ML) tree using the command “-i input-bootstrap-o output”. Second, the -i input -bootstrap -k 1000 -m migration events -o output command is used to predict migration events. Genetic introgression was also analysed by the D-statistic (ABBA-BABA test) using AdmixTools software [56, 57].

PLINK (v1.90b6.20) was used to detect ROHs (settings: --homozyg-window-snp 100 --homozyg-window-het 2 --homozyg-window-missing 5 --homozyg-snp 100 --homozyg-kb 100 --homozyg-density 10 --homozyg-gap 100) [58].

Dynamic changes in the effective population size were inferred using the PSMC program (v0.6.5-r67) [59], with a mutation rate of 2.0 × 10^− 9^ and a generation time of 2 years [60].

### Selective sweep analysis

*F*_*ST*_ indicates the degree of group differentiation. The larger the value is, the higher the degree of group differentiation and the higher the degree of selection [61]. Using *F*_*ST*_ and θπ ratios to detect selective sweep regions had many applications in the genomics analysis of plants and animals and has proved to be a powerful method [25, 62–64]. *F*_*ST*_ and θπ ratios were calculated using VCFtools with a 20-kb sliding window and 10-kb steps for comparisons between individual groups [65]. We then filtered out windows with fewer than 10 SNPs in the *F*_*ST*_ and θπ results. We restricted the scanning for divergent regions description to the significance level of *F*_*ST*_ and π ratio using the top 5% highest log2 (θπ·wild/θπ·Cultivated) and top 5% highest *F*_*ST*_ and at the top 0.1% highest *F*_*ST*_ because these windows represent the extremes of the distribution. They contain 41 and 62 genes, respectively. Manually weeded out genes that could not be annotated. Gene Ontology (GO) enrichment analyses were performed using the DAVID program (https://david.ncifcrf.gov/) and g: profiler (https://biit.cs.ut.ee/gprofiler/) with significant threshold Q value ≤ 0.05.

## Electronic supplementary material

Below is the link to the electronic supplementary material.

Supplementary Material 1

Supplementary Material 2

Supplementary Material 3

Supplementary Material 4

Supplementary Material 5

Supplementary Material 6

Supplementary Material 7

Supplementary Material 8

Supplementary Material 9

Supplementary Material 10

Supplementary Material 11

Supplementary Material 12

Supplementary Material 13

Supplementary Material 14

Supplementary Material 15

Supplementary Material 16

Supplementary Material 17

Supplementary Material 18

Supplementary Material 19

Supplementary Material 20

## Data Availability

All the sequences reported in this study are deposited into National Center for Biotechnology Information SRA Database, under the BioProject PRJNA902712 (https://www.ncbi.nlm.nih.gov/bioproject/PRJNA902712).

## List of Abbreviations

SNP: Single-nucleotide polymorphism
PCA: Principal component analysis
NJ tree: Neighbour-joining tree
*F*_*ST*_: F-statistics index
Gene Ontology: GO
CV: Coefficient of variation
ROH: Runs of homozygosity
Ts/Tv: Ratio of transitions/transversions
