# Supplementary figures and images for: Resequencing of Rosa rugosa accessions revealed the history of population dynamics, breed origin, and domestication pathways
Source: BMC Plant Biol. 2023 May 4;23:235. doi: 10.1186/s12870-023-04244-5 (PMC10158352; doi:10.1186/s12870-023-04244-5)

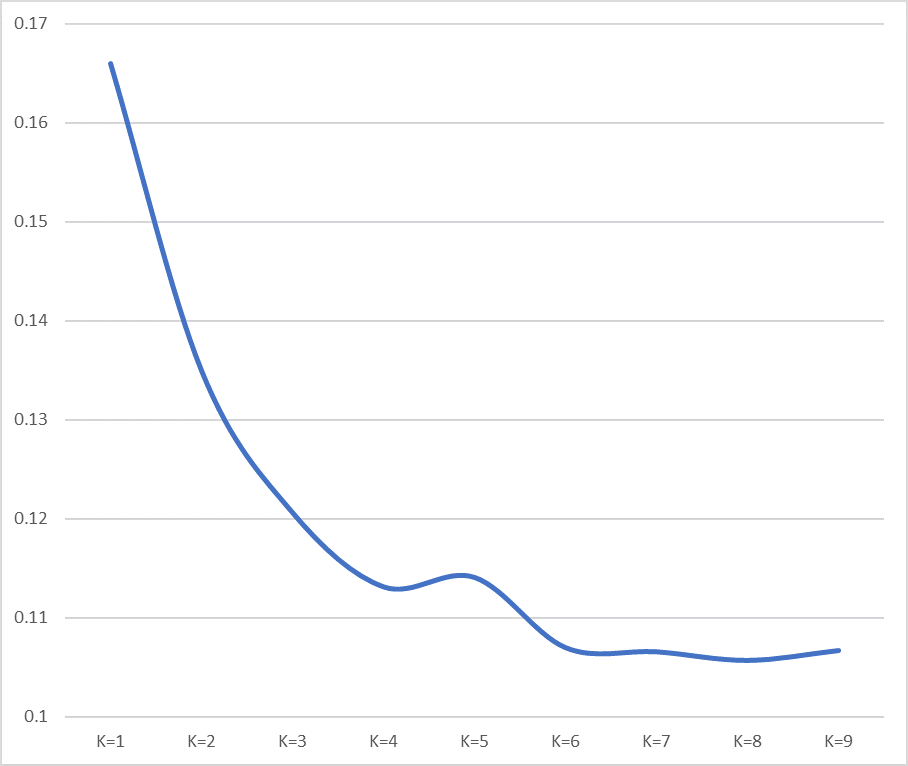

Supplement: Supplementary file 1 — Supplementary Material 1 [file 12870_2023_4244_MOESM1_ESM.png]

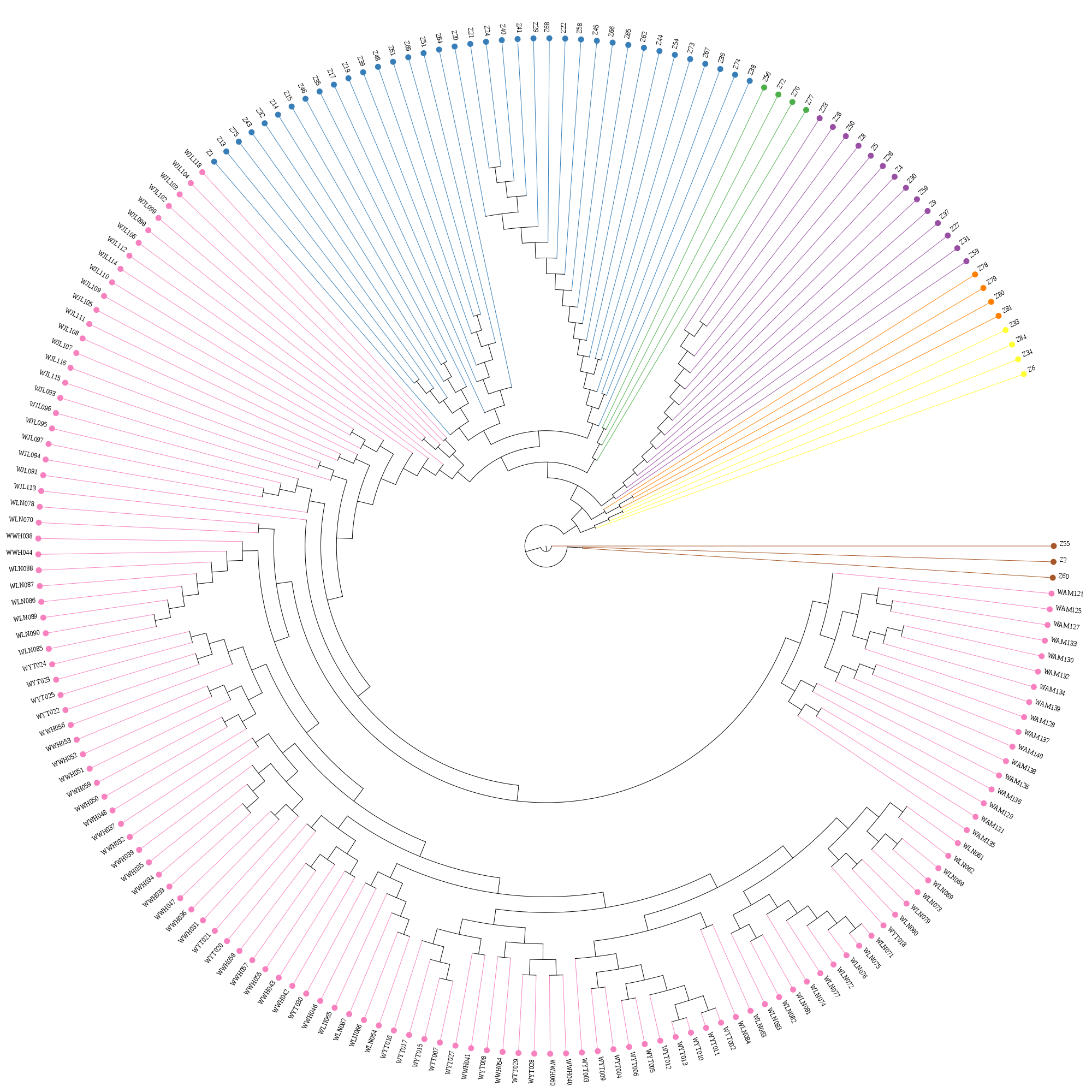

Supplement: Supplementary file 2 — Supplementary Material 2 [file 12870_2023_4244_MOESM2_ESM.png]

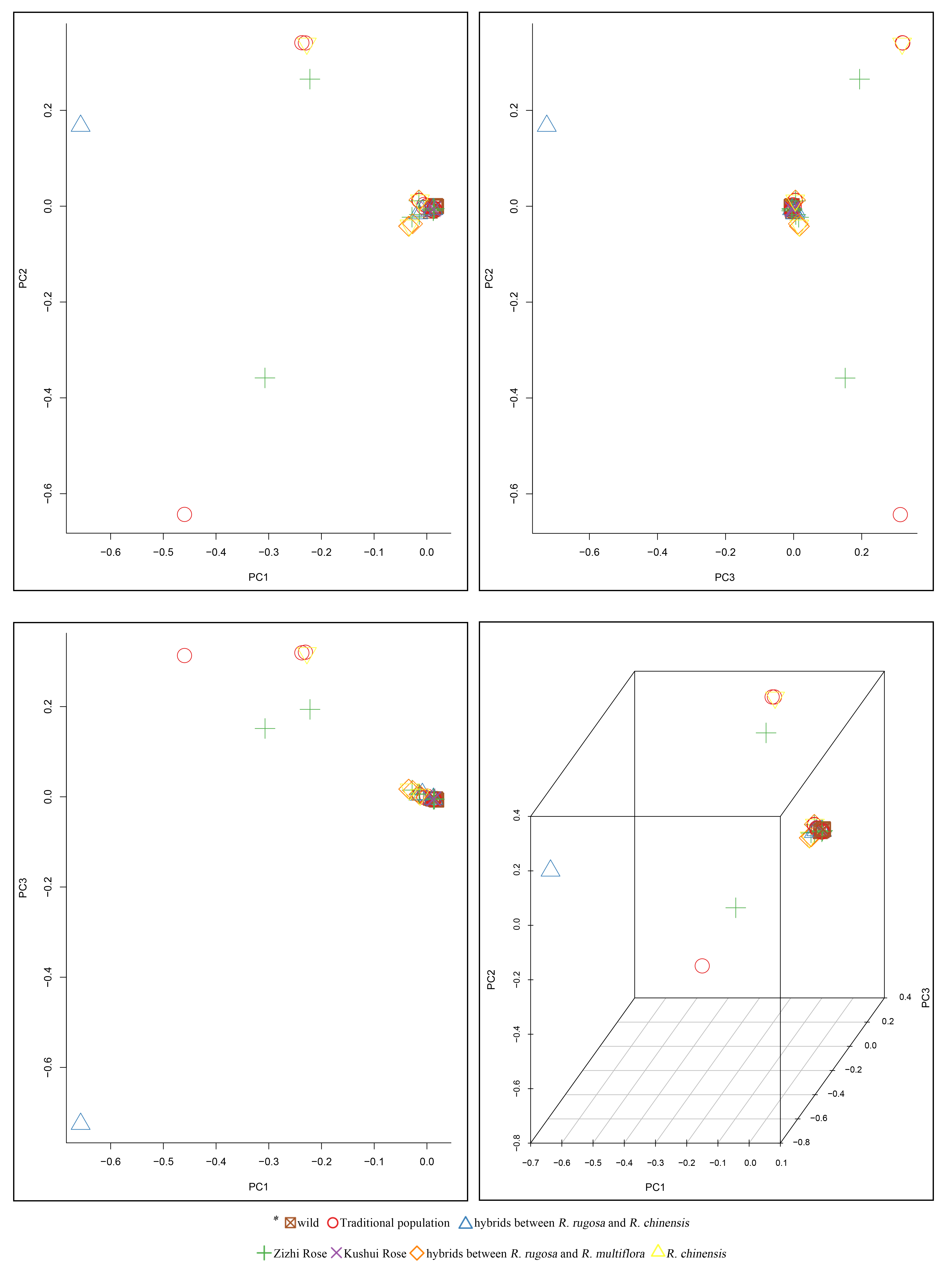

Supplement: Supplementary file 3 — Supplementary Material 3 [file 12870_2023_4244_MOESM3_ESM.jpg]

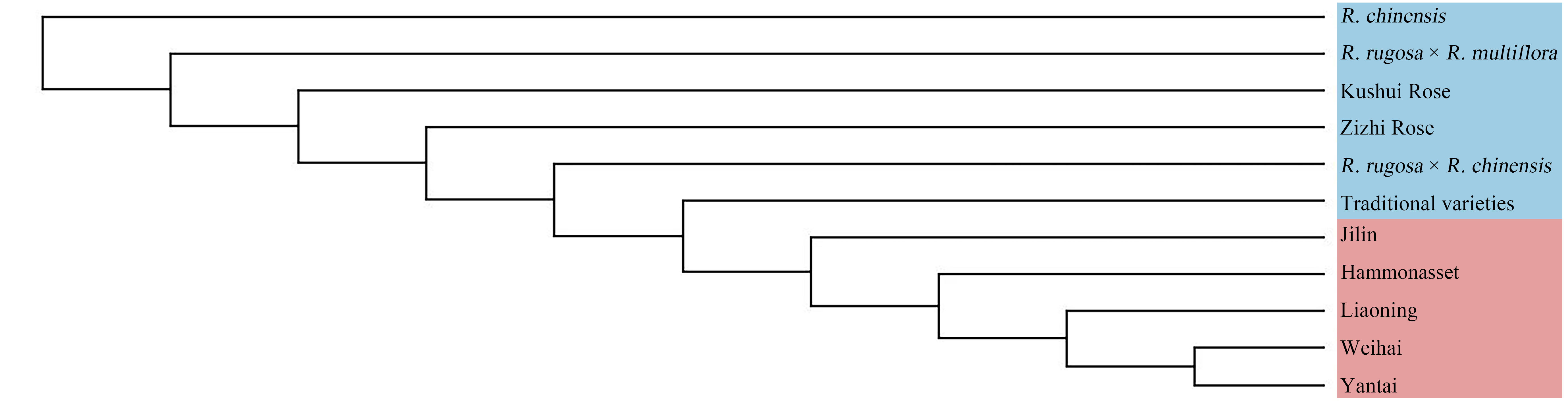

Supplement: Supplementary file 4 — Supplementary Material 4 [file 12870_2023_4244_MOESM4_ESM.jpg]

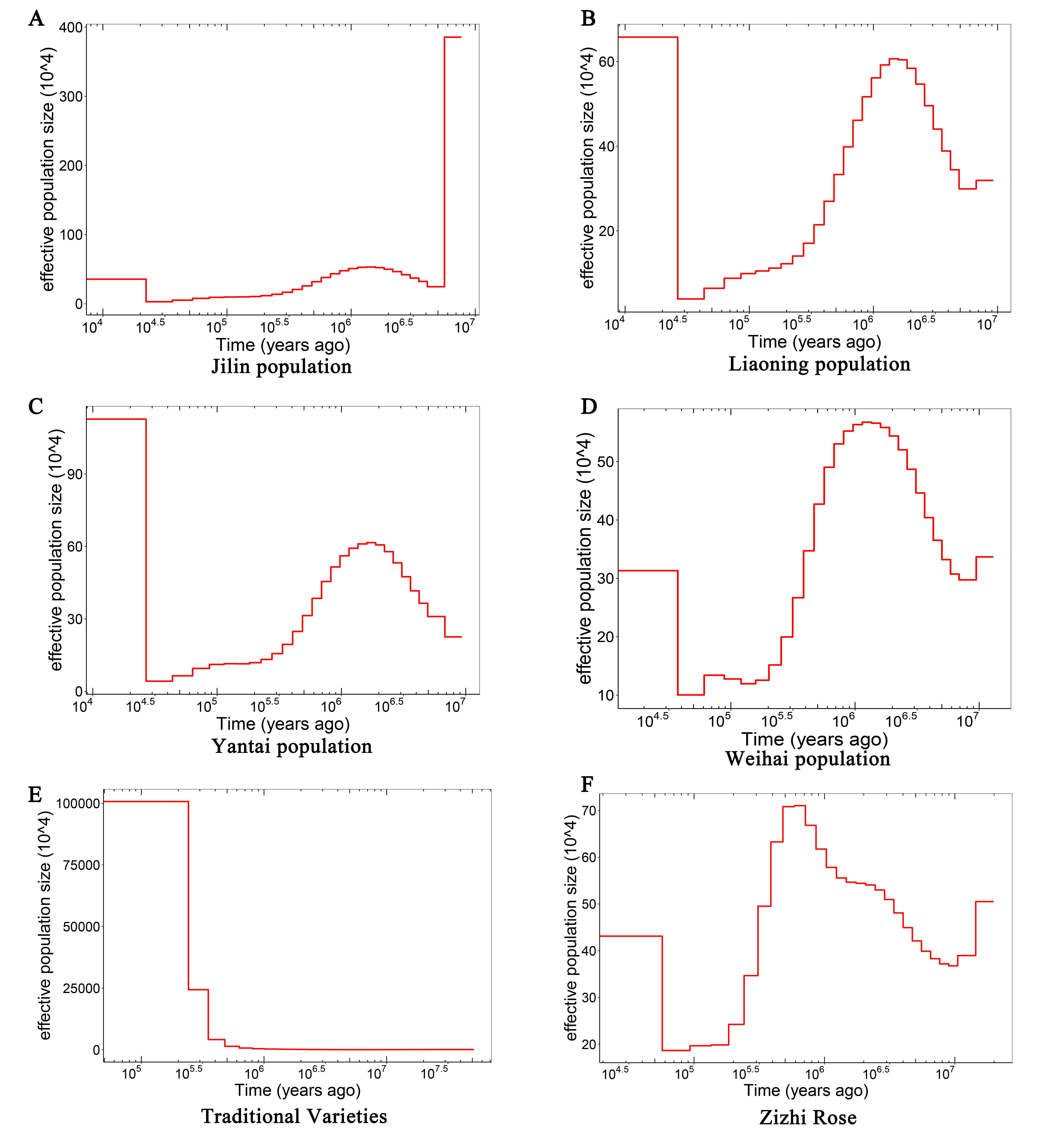

Supplement: Supplementary file 5 — Supplementary Material 5 [file 12870_2023_4244_MOESM5_ESM.jpg]

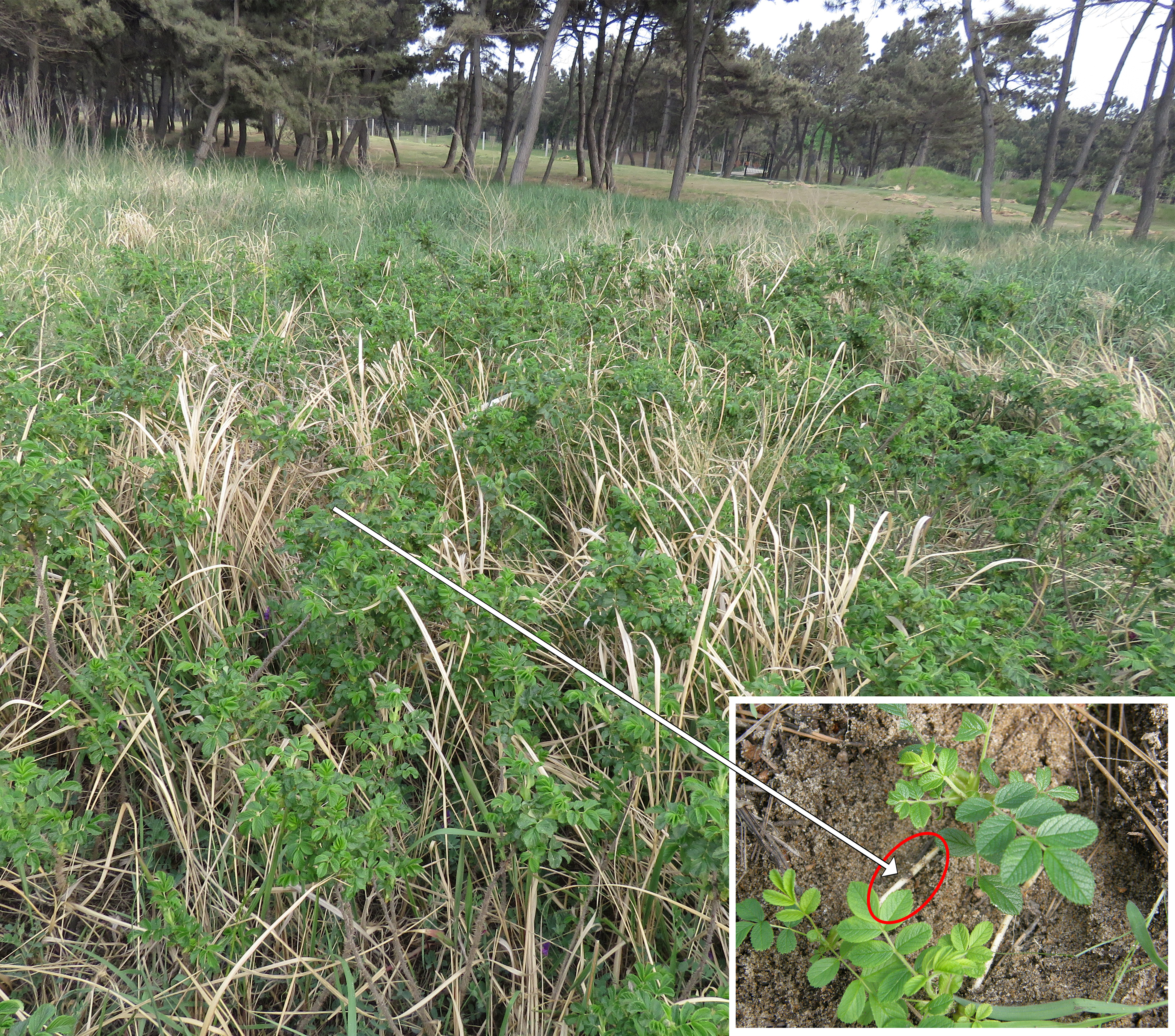

Supplement: Supplementary file 6 — Supplementary Material 6 [file 12870_2023_4244_MOESM6_ESM.jpg]
