# Supplementary material for: Resequencing of Rosa rugosa accessions revealed the history of population dynamics, breed origin, and domestication pathways
Source: BMC Plant Biol. 2023 May 4;23:235. doi: 10.1186/s12870-023-04244-5 (PMC10158352; doi:10.1186/s12870-023-04244-5)
Supplement: Supplementary file 18 — Supplementary Material 18 [file 12870_2023_4244_MOESM18_ESM.docx]

## Additional files

Additional file 1: Table S1. Samples of wild and cultivated roses resequenced in this study.

Additional file 2: Table S2. Resequencing descriptive statistics data for the 191 samples.

Additional file 3: Table S3. Genome mapping and coverage information for all accessions analysed in this study.

Additional file 4: Table S4. Number and distribution of SNPs for each individual.

Additional file 5: Table S5. Summary of whole genome SNP distribution for different groups.

Additional file 6: Table S6. The average polymorphism in different groups.

Additional file 7: Table S7. Pairwise *F_ST_* values among the following groups.

Additional file 8: Table S8. BABA-ABBA estimates.

Additional file 9: Table S9. Inbreeding coefficients and ROH information.

Additional file 10: Table S10. Selected regions by top 1% highest log2 (θπwild/θπdomesticated) and top 5% *F_ST_*.

Additional file 11: Table S11. Selected regions by top 0.1% *F_ST_*.

Additional file 12: Table S12. Genes in the selected sweep regions by top 1% highest log2 (θπ·wild/θπ·Cultivated) and top 5% highest *F_ST_*.

Additional file 13: Table S13. Genes in the selected sweep regions by the top 0.1% highest *F_ST_.*

Additional file 14: Fig. S1. Phylogenetic tree of 191 *R. rugosa* accessions.

Additional file 15: Fig. S2. Coefficient of variation (CV) error value.

Additional file 16: Fig. S3. Principal component analysis (PCA) of 191 *R. rugosa* accessions.

Additional file 17: Fig. S4. Maximum likelihood phylogenetic tree of the *R. rugosa* population.

Additional file 18: Fig. S5. Historical population size dynamics of wild and some cultivated populations (A. Jilin population; B. Liaoning population; C. Yantai population; D. Weihai population; E. traditional varieties; F. Zizhi Rose).

Additional file 19: Fig. S6. Wild *R. rugosa* propagates by tillering.
